# IMMUNOREACT 4: Peritumoral Microenvironment Associated with Anastomotic Leaks After Surgery for Rectal Cancer

**DOI:** 10.3390/cancers18040571

**Published:** 2026-02-09

**Authors:** Ottavia De Simoni, Melania Scarpa, Francesco Cavallin, Andromachi Kotsafti, Francesco Marchegiani, Astghik Stepanyan, Gaia Tussardi, Antonio Rosato, Gaya Spolverato, Imerio Angriman, Emanuele Damiano Luca Urso, Cesare Ruffolo, Luca Maria Saadeh, Isacco Maretto, Quoc Riccardo Bao, Silvia Negro, Chiara Vignotto, Luca Facci, Giorgio Rivella, Antonella D’Angelo, Anna Matteazzi, Francesca Galuppini, Vincenza Guzzardo, Roberta Salmaso, Valerio Pellegrini, Stefano Brignola, Carlotta Ceccon, Tommaso Stecca, Anna Pozza, Marco Massani, Pierluigi Pilati, Mario Gruppo, Boris Franzato, Ivana Cataldo, Giuseppe Portale, Chiara Cipollari, Matteo Zuin, Licia Laurino, Luca Dal Santo, Giovanni Pirozzolo, Alfonso Recordare, Lavinia Ceccarini, Michele Antoniutti, Laura Marinelli, Alberto Brolese, Mattia Barbareschi, Giovanni Bertalot, Monica Ortenzi, Mario Guerrieri, Maurizio Zizzo, Lorenzo Dell’Atti, Silvio Guerriero, Alessandra Piccioli, Giulia Pozza, Mario Godina, Isabella Mondi, Daunia Verdi, Corrado Da Lio, Giulia Noaro, Roberto Cola, Giovanni Bordignon, Roberto Merenda, Giulia Becherucci, Laura Gavagna, Salvatore Candioli, Giovanni Tagliente, Umberto Tedeschi, Dario Parini, Beatrice Salmaso, Gianluca Businello, Loretta Di Cristoforo, Francesca Bergamo, Andrea Porzionato, Federico Scognamiglio, Romeo Bardini, Salvatore Pucciarelli, Marco Agostini, Valentina Chiminazzo, Dario Gregori, Barbara Di Camillo, Ignazio Castagliuolo, Angelo Paolo Dei Tos, Matteo Fassan, Marco Scarpa

**Affiliations:** 1 Surgical Oncology of Digestive Tract Unit, Veneto Institute of Oncology IOV-IRCCS, 35128 Padova, Italy; pierluigi.pilati@iov.veneto.it (P.P.); mario.gruppo@iov.veneto.it (M.G.); boris.franzato@iov.veneto.it (B.F.); 2 Immunology and Molecular Oncology Diagnostics, Veneto Institute of Oncology IOV-IRCCS, 35128 Padova, Italy; melania.scarpa@iov.veneto.it (M.S.); andromachi.kotsafti@iov.veneto.it (A.K.); antonio.rosato@unipd.it (A.R.); 3 Independent Statistician, 36020 Solagna, Italy; 4 Unit of Colorectal and Digestive Surgery, Beaujon Hospital, 92110 Paris, France; 5 Chirurgia Generale 3, Azienda Ospedale Università di Padova, 35128 Padova, Italy; stepanyan.astgh@gmail.com (A.S.); gaiatussardi@gmail.com (G.T.); cesare.ruffolo@aopd.veneto.it (C.R.); luca.saadeh@gmail.com (L.M.S.); isacco.maretto@aopd.veneto.it (I.M.); quocriccardo.bao@gmail.com (Q.R.B.); silvia.negrosn01@gmail.com (S.N.); chiara.vignotto.cv@gmail.com (C.V.); lucafacci1@gmail.com (L.F.); giorgio.rivella@gmail.com (G.R.); antonella.dangelo@aopd.veneto.it (A.D.); anna.matteazzi@aopd.veneto.it (A.M.); federico.scognamiglio@ubep.unipd.it (F.S.); 6 Dipartimento di Scienze Chirurgiche, Oncologiche e Gastroenterologiche (DISCOG), University of Padova, 35128 Padova, Italy; gaya.spolverato@unipd.it (G.S.); imerio.angriman@unipd.it (I.A.); edl.urso@unipd.it (E.D.L.U.); romeo.bardini@unipd.it (R.B.); puc@unipd.it (S.P.); m.agostini@unipd.it (M.A.); 7 Pathology Unit, University of Padova, 35128 Padova, Italy; francesca.galuppini@unipd.it (F.G.); vincenza.guzzardo@unipd.it (V.G.); roberta.salmaso@unipd.it (R.S.); stefano.brignola@aulss2.veneto.it (S.B.); angelo.deitos@unipd.it (A.P.D.T.); 8 Pathology Unit, Ospedale Ca’Foncello, AULSS 2 Marca Trevigiana, 31100 Treviso, Italy; valeriopellegrini1995@gmail.com (V.P.); carlotta.ceccon@unipd.it (C.C.); matteo.fassan@gmail.com (M.F.); 9 General Surgery Unit III, Ospedale Ca’ Foncello, AULSS 2 Marca Trevigiana, 31100 Treviso, Italy; tomaz86@gmail.com (T.S.); annapozza@yahoo.it (A.P.); marco.massani@aulss2.veneto.it (M.M.); 10 Oncological Anatomy and Histology Unit, Veneto Institute of Oncology IOV-IRCCS, 35128 Padova, Italy; ivana.cataldo@iov.veneto.it; 11 General Surgery Unit, Ospedale Alto Vicentino, Azienda ULSS 7 Pedemontana, 36014 Santorso, Italy; giuseppe.portale@aulss7.veneto.it; 12 General Surgery Unit, Ospedale di Cittadella, Azienda ULSS 6 Euganea, 35013 Cittadella, Italy; chiara.cipollari@aulss6.veneto.it (C.C.); matteo.zuin@aulss6.veneto.it (M.Z.); giovanni.pirozzolo@gmail.com (G.P.); alfonso.recordare@aulss3.veneto.it (A.R.); 13 Pathology Unit, Ospedale Dell’Angelo, Azienda ULSS 3 Serenissima, 30174 Venezia, Italy; licia.laurino@aulss3.veneto.it (L.L.); luca.dalsanto@aulss3.veneto.it (L.D.S.); 14 General Surgery Unit, Ospedale San Bassiano, Azienda ULSS 7 Pedemontana, 36061 Bassano del Grappa, Italy; lavinia.ceccarini@aulss7.veneto.it (L.C.); michele.antoniutti@aulss7.veneto.it (M.A.); 15 General Surgery Unit, Ospedale Santa Chiara, Azienda Provinciale Socio Sanitaria di Trento, 38122 Trento, Italy; laura.marinelli@apss.tn.it (L.M.); alberto.brolese@apss.tn.it (A.B.); 16 Pathology Unit, Ospedale Santa Chiara, University of Trento c/o Azienda Provinciale Socio Sanitaria di Trento, 38122 Trento, Italy; mattia.barbareschi@apss.tn.it (M.B.); giovanni.bertalot@apss.tn.it (G.B.); 17 General Surgery Unit, Ospedali Riuniti, 60126 Ancona, Italy; monica.ortenzi@gmail.com (M.O.); mario.guerrieri@ospedaliriuniti.marche.it (M.G.); 18 Surgical Oncology Unit, Azienda Unità Sanitaria Locale-IRCCS di Reggio Emilia, 42123 Reggio Emilia, Italy; maurizio.zizzo@ausl.re.it (M.Z.); lorenzo.dellatti@ausl.re.it (L.D.); 19 General Surgery Unit, Ospedale di Fermo, ASUR 4, 63900 Fermo, Italy; silguerri@gmail.com (S.G.); alessandrapiccioli@gmail.com (A.P.); 20 Bariatric Surgery Unit, Azienda Ospedale Università di Padova, 35128 Padova, Italy; giulia.pozza@aopd.veneto.it; 21 General Surgery Unit, Ospedale di Dolo, Azienda ULSS 3, 30031 Venezia, Italy; mario.godina@aulss3.veneto.it; 22 General Surgery Unit, Ospedale di Mirano, Azienda ULSS 3 Serenissima, 30035 Venezia, Italy; isabella.mondi@aulss3.veneto.it (I.M.); daunia.verdi@aulss3.veneto.it (D.V.); corrado.dalio@aulss3.veneto.it (C.D.L.); 23 General Surgery Unit, Ospedali Riuniti Padova Sud Madre Teresa di Calcutta, Azienda ULSS 6 Euganea, 35043 Padova, Italy; giulia.noaro@aulss6.veneto.it (G.N.); roberto.cola@aulss6.veneto.it (R.C.); 24 General Surgery Unit, Ospedale Santi Giovanni e Paolo, Azienda ULSS 3 Serenissima, 30122 Venezia, Italy; giovanni.bordignon@aulss3.veneto.it (G.B.); roberto.merenda@aulss3.veneto.it (R.M.); 25 General Surgery Unit, Ospedale San Martino, Azienda ULSS 1 Dolomiti, 32100 Belluno, Italy; giulia.becherucci@aulss1.veneto.it (G.B.); laura.gavagna@aulss1.veneto.it (L.G.); salvatore.candioli@aulss1.veneto.it (S.C.); 26 General Surgery Unit, Ospedale Dell’Angelo, Azienda ULSS 3 Serenissima, 30174 Venezia, Italy; giovanni.tagliente@aulss3.veneto.it; 27 General Surgery Unit, Casa di Cura di Abano, 35031 Padova, Italy; utedeschi@casacura.it; 28 General Surgery Unit, Ospedale Santa Maria della Misericordia, AULSS 5 Polesana, 45100 Rovigo, Italy; dario.parini@aulss5.veneto.it (D.P.); beatrice.salmaso@aulss5.veneto.it (B.S.); 29 Pathology Unit, Ospedale Santa Maria della Misericordia, AULSS 5 Polesana, 45100 Rovigo, Italy; gianluca.businello@aulss5.veneto.it; 30 General Surgery Unit, Ospedale Grassi, ASL ROMA 3, 00122 Ostia, Italy; loretta.dicristofaro@aslroma3.it; 31 Medical Oncology 1, Veneto Institute of Oncology IOV-IRCCS, University of Padova, 35128 Padova, Italy; francesca.bergamo@iov.veneto.it; 32 Department of Neurosciences (DNS), University of Padova, 35128 Padova, Italy; andrea.porzionato@unipd.it; 33 Health Research Institute of the Principality of Asturias (ISPA), 33011 Oviedo, Spain; valentina.chiminazzo@unipd.it; 34 Department of Cardiac, Thoracic, Vascular Sciences and Public Health, University of Padova, 35128 Padova, Italy; dario.gregori@unipd.it; 35 Information Engineering (DEI), University of Padova, 35128 Padova, Italy; barbara.dicamillo@unipd.it; 36 Department of Molecular Medicine (DMM), University of Padova, 35128 Padova, Italy; ignazio.castagliuolo@unipd.it

**Keywords:** rectal cancer, anastomotic leak, immune surveillance markers

## Abstract

**Background:** Anastomotic leaks (ALs) remain a critical complication after rectal cancer surgery. Emerging evidence suggests that local immune dysregulation may play a key role in anastomotic healing. We investigated the immune microenvironment of histologically normal, tumor-adjacent rectal mucosa—a tumor-conditioned field—as a potential substrate for AL predisposition. **Methods:** IMMUNOREACT 4 is a sub-analysis of the IMMUNOREACT project (clinicaltrials.gov NCT04915326 and NCT04915326), a multicenter translational study evaluating immune features of histologically normal, tumor-adjacent rectal mucosa of patients undergoing colorectal anastomosis. A prospective cohort (n = 121) was analyzed using flow cytometry, in addition to a retrospective cohort (n = 262) using immunohistochemistry. Immune markers of epithelial activation and lymphocyte subsets were compared between patients with and without postoperative ALs. Exploratory predictive models combining immune and clinical variables were developed and evaluated using discrimination, calibration and decision curve analyses. **Results:** At flow cytometry, the CK^+^HLAabc^+^ MFI (AUC 0.66, 95% CI 0.52–0.80), CD8^+^CD38^+^ cell rate (AUC 0.65, 95% CI 0.52–0.78) and CD3^+^CTLA4^+^ cell rate (AUC 0.65, 95% CI 0.51–0.80) showed moderate predictive potential for ALs. In immunohistochemistry, CD3^+^ (AUC 0.57, 95% CI 0.54–0.60), CD8^+^ (AUC 0.57, 95% CI 0.52–0.62), CD8β^+^ (AUC 0.59, 95% CI 0.53–0.65) and Tbet^+^ (AUC 0.60, 95% CI 0.56–0.64) showed some predictive ability for ALs. The model including CD8β^+^, the BMI, neutrophile/lymphocyte ratio and tumor location had an AUC of 0.67 (95% CI 0.62–0.72). **Conclusions:** Immune activation within histologically normal, tumor-adjacent rectal mucosa—characterized by epithelial HLA upregulation and cytotoxic or Th1 T cell infiltration—is associated with postoperative ALs. Although predictive accuracy is limited, these findings support the concept that a tumor-conditioned immune microenvironment may predispose patients to impaired anastomotic healing. Integration of mucosal immune profiling with clinical variables represents a promising exploratory approach that warrants further prospective validation.

## Simple Summary

In this multicenter study of 363 patients, we provide the first evidence that immune profiling of histologically normal, tumor-adjacent mucosa may identify patients at risk for anastomotic leaks (ALs) after rectal surgery. Among the markers assessed, only CD3^+^, CD8^+^, CD8β^+^ and Tbet^+^ showed predictive value, suggesting that the mucosa intended for anatomosis may already be compromised by hypoxia, extensive cell death, autoimmune inflammation or infection. We then explored combinations of clinical and immunological markers to obtain predictive models of ALs. Models including CD8^+^ (or CD8β^+^), BMI, neutrophil-to-lymphocyte ratio and tumor location achieved ~70% accuracy. Although the predictive accuracy of our models remains relatively limited, combining immune microenvironmental data from the healthy mucosa with clinical variables may represent a promising approach to identifying patients at risk of ALs and deserves further testing in clinical practice.

## 1. Introduction

Anastomotic leaks (ALs) remain a critical complication following rectal cancer surgery, with reported morbidity and mortality rates ranging from 4% to 29% [1]. Importantly, symptomatic ALs are a recognized risk factor for disease recurrence in patients with rectal adenocarcinoma [2]. The TENTACLE-Rectum study further highlighted the long-term burden of this complication, reporting a one-year stoma-free survival rate of only 43.7–45.0% in patients who developed ALs [3]. In addition, if an AL necessitates reoperation, then the patient-surgeon relationship may be irreversibly compromised [4].

To mitigate the clinical consequences of ALs, several preventive strategies have been adopted, including selective creation of diverting ileostomy, particularly in patients considered at high risk, such as those with extremely low anastomoses [5]. However, risk stratification remains imperfect. A large international cohort study involving 2470 patients showed substantial variability in postoperative management after an AL, with 16% undergoing salvage surgery, 62% having passive drainage, 11% having active drainage, and 11% having no fecal diversion [3]. These data highlight the need for improved understanding of the biological mechanisms underlying ALs, beyond purely technical or ischemic explanations. Insufficient vascular supply is regarded as the main cause of ALs after rectal resection [6]. However, the etiology of ALs is clearly multifactorial and incompletely understood. Patient-related risk factors include being of the male sex, a higher American Society of Anesthesiologist (ASA) score, comorbidities and an elevated BMI [7,8,9,10,11,12]. Intra-operative factors such as longer operative times and excessive blood loss have also been linked to increased leakage rates [12]. Several predictive models have therefore been developed to guide selective use of protective ileostomy. For instance, a predictive model after transanal total mesorectal excision incorporating sex, BMI, smoking status, diabetes and tumor size was proposed by Penna et al. [13]. Li et al. reported that neoadjuvant chemoradiotherapy, blood loss >50 mL and intersphincteric predicted releakage after stoma closure [14]. Other models have included postoperative inflammatory markers measured in blood or in the perianastomotic drain fluid [15,16]. Rutegard et al. designed a clinical prediction model for AL after rectal surgery, including being of the male sex, a BMI >30 kg/m^2^ and radiotherapy, which achieved an area under the curve (AUC) of 0.64 (95% CI 0.62–0.65) [17]. Notably, this model was conceived for prospective application in randomized clinical trials, similar to the intended use of the models in our study.

The integration of inflammatory markers into clinical scores is supported by a strong biological rationale. For example, the postoperative neutrophil-to-lymphocyte ratio (NLR) has been shown to be significantly higher in patients who develop ALs compared with those who do not [18]. Several studies found significantly higher levels of TNF-α and IL-6 in the drained fluids of patients with anastomotic leakage during the first three postoperative days [19]. Conversely, lower preoperative levels of macrophage inflammatory protein-1 α (MIP-1α), monocyte chemotactic protein-1 (MCP-1), IL-8, FGF2 and G-CSF were associated with subsequent ALs [20].

Despite these advances, the role of the immune microenvironment of histologically normal, tumor-adjacent rectal mucosa has never been investigated as a predictor of ALs following rectal resection. Although histologically unremarkable, tumor-adjacent mucosa is increasingly recognized as biologically altered by the presence of cancer, consistent with the concept of a tumor-conditioned field effect. This microenvironment may exhibit immune activation, epithelial stress responses and inflammatory priming that are not captured by conventional histopathology but may critically influence anastomotic healing.

The central hypothesis of the IMMUNOREACT 4 study is that immune activation within histologically normal, tumor-adjacent rectal mucosa reflects a preexisting biological vulnerability that predisposes patients to postoperative ALs. By integrating immune profiling using flow cytometry and immunohistochemistry with clinical variables, this study aims to explore the biological mechanisms linking local immune dysregulation to impaired anastomotic healing and assess whether these features may contribute to exploratory predictive models of AL risk.

## 2. Materials and Methods

### 2.1. Study Design

IMMUNOREACT 4 is a sub-analysis of the IMMUNOREACT project (clinicaltrials.gov NCT04915326 and NCT04915326), enrolling rectal cancer patients undergoing radical surgery with colorectal anastomosis. Clinical, operative and pathological records were retrieved. An AL was defined as a defect in the intestinal wall at the anastomotic site leading to communication between intra- and extra-luminal compartments, as specified by the International Study group of Rectal Cancer [21]. To assess the gravity of AL the classification described by Soeters et al. was used which includes four progressive severity grades [22]. The aim was to investigate whether immune infiltration within the histologically normal, tumor-adjacent rectal mucosa could predict postoperative ALs. Tissue samples were obtained from the proximal resection margin at the intended anastomotic site at least 5 cm from the macroscopic tumor’s edge. Fresh tissue was collected immediately after surgical resection and divided into portions for flow cytometry (processed within two hours) and formalin fixation for immunohistochemistry. A board-certified gastrointestinal pathologist reviewed H&E sections from each sample to confirm histologically normal mucosa without evidence of tumor infiltration, high-grade dysplasia or active inflammation. Only patients with complete perioperative and follow-up information were included. This multicenter project was performed according to the principles of the Declaration of Helsinki, and it was approved by the ethical committees of the involved hospitals.

In the retrospective step of the IMMUNOREACT 4 study, a panel of immunohistochemistry markers (CD3 (pan T cell marker), CD4 (T helper marker), CD8 (cytotoxic marker), CD8beta (marker of cytotoxic activation), Tbet (T-bet, or T box expressed in T cells, a Th1 marker), FoxP3 (forkhead box P3, a Treg marker) and PD-L1 (programmed death ligand 1) of immune surveillance was tested on the histologically normal, tumor-adjacent rectal mucosa. Immunohistochemistry quantification was performed via manual counting on five high-power fields (40× magnification) selected from areas with optimal tissue preservation and staining quality. A single pathologist blinded to the clinical outcomes quantified the positive cells within both the intraepithelial and lamina propria compartments. Cell densities are reported as cells per high-power field. The interobserver agreement coefficient was 0.87. Detailed antibody specifications are provided in Supplementary Table S1.

For the prospective part of the IMMUNOREACT 4 study, a panel of flow cytometry markers (CD3^+^ CTLA-4^+^ (pan T cell inhibitor marker), CD4^+^CD25^+^FoxP3^+^ (Treg marker), CD8^+^CD28^+^ and CD8^+^CD38^+^ (activated cytotoxic markers), CK^+^CD80^+^, CK^+^CD86^+^ and CK^+^HLAabc^+^ (markers of epithelial cells acting as antigen presenting cells) of immune surveillance was tested on the histologically normal, tumor-adjacent rectal mucosa. Detailed antibody specifications are provided in Supplementary Table S2.

**Figure 1:**
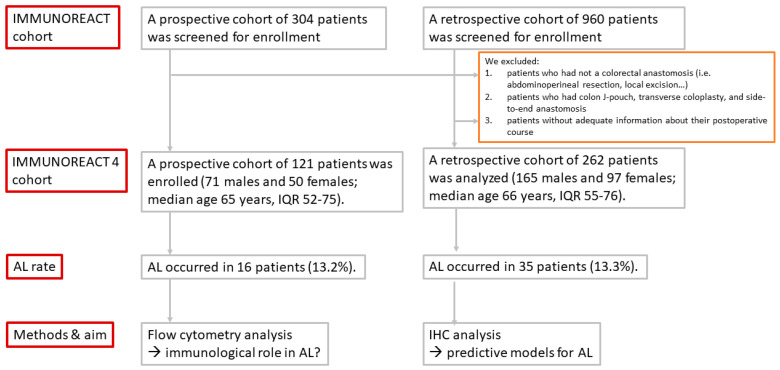
Study design with the selection of the prospective and retrospective cohorts.

Immune markers within the histologically normal, tumor-adjacent rectal mucosa were compared between patients who developed or did not develop a postoperative AL, and predictive models were then created. We excluded patients who did not have AL, such as surgical procedures that do not involve colorectal anastomosis (i.e., abdominoperineal resection, Hartman procedures or local excision), those who had colon J-pouches, transverse coloplasty, and side-to-end anastomosis and those who did not have adequate information about their postoperative course. The study design is shown in Figure 1.

### 2.2. Statistical Analysis

Numerical data were summarized as the median and interquartile range (IQR), while categorical data were summarized as the absolute and relative frequencies (percentage). The expression profile was compared between patients with or without ALs using the Mann–Whitney test. In both the prospective and retrospective cohorts, the discrimination of cases with ALs was assessed with the area under the receiver operating characteristic curve (AUC) with a 95% confidence interval (CI) for each molecular predictor of an AL. Sensitivity (with 95% CI) at a fixed specificity (0.90) and specificity (with 95% CI) at a fixed sensitivity (0.90) were calculated to compare the molecular predictor of ALs. In the retrospective cohort, the molecular predictors of ALs had missing data; hence, multiple imputation by chained equations (MICE) was used to manage the missing data. Missingness at random (MAR) was reasonably assumed; five imputed datasets were produced; and the imputations were based on all variables in the original dataset. The imputed and observed data matched up well and suggested the plausibility of imputations (Supplementary Figure S1). A sensitivity analysis on the original dataset before multiple imputation is also provided in the Supplementary Materials. In the retrospective cohort, we estimated a logistic model for the occurrence of ALs including the molecular predictor of an AL, with the 95% CI of their AUC not including 0.50. Further models including one of these variables and a set of clinically relevant variables (BMI, neutrophil/lymphocyte ratio and tumor location) were also estimated. In addition, the model by Rutegard et al. [17] (including male sex, BMI > 30 kg/m^2^ and radiotherapy) was also evaluated. For each model, we calculated the AUC, sensitivity at a fixed specificity (0.90) and specificity at a fixed sensitivity (0.90) with their 95% CI. The calibration was assessed using the calibration curve, and the clinical utility was evaluated using the decision curve analysis to identify which patients should be anticipated as prone to the occurrence of ALs. The predictive models were developed for exploratory purposes and aimed at identifying tissue-level biological vulnerability rather than providing clinically actionable risk prediction tools. The statistical significance was set at 5%. Statistical analysis was carried out using R 4.4 (R Foundation for Statistical Computing, Vienna, Austria) [23].

## 3. Results

### 3.1. Epithelial Cells and Intramucosal Lymphocytes in Histologically Normal, Tumor-Adjacent Rectal Mucosa Associated with ALs

In the first step of the IMMUNOREACT 4 study, we investigated the interplay between epithelial cells and intramucosal lymphocytes of the histologically normal, tumor-adjacent rectal mucosa in predisposing to ALs. A prospective cohort of 121 patients was enrolled (71 males and 50 females; median age of 65 years, IQR 52–75). ALs occurred in 16 patients (13.2%). The demographics and clinical characteristics are summarized in Table 1 and the Supplementary Materials.

The activation profile of the epithelial cells and intramucosal lymphocytes of the histologically normal, tumor-adjacent rectal mucosa, as assessed by flow cytometry, is summarized in Table 2.

**Figure 2:**
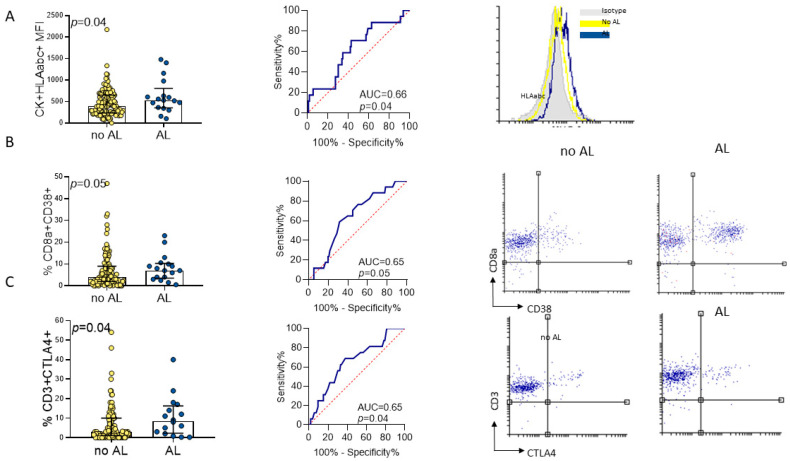
(**A**) CK^+^HLAabc^+^ MFI in healthy rectal mucosa as predictor of anastomotic leaks. (**B**) CD8^+^CD38^+^ cell rate in histologically normal, tumor-adjacent rectal mucosa as predictor of anastomotic leaks. (**C**) CD3^+^CTLA4^+^ cell in healthy rectal mucosa as predictor of anastomotic leaks.

Patients who developed ALs showed significantly higher CK^+^HLAabc^+^ MFIs (median 546 [IQR 445–719] vs. 407 [IQR 247–642]; *p* = 0.04), CD8^+^CD38^+^ cell rates (median 8 (4–10) vs. median 4 (2–8), *p =* 0.05) and CD3^+^CTLA4^+^ cell rates (median 8 [IQR 3–15] vs. 3 [IQR 1–10]; *p =* 0.04) compared with those without ALs (Figure 2 and Table 2).

**Figure 3:**
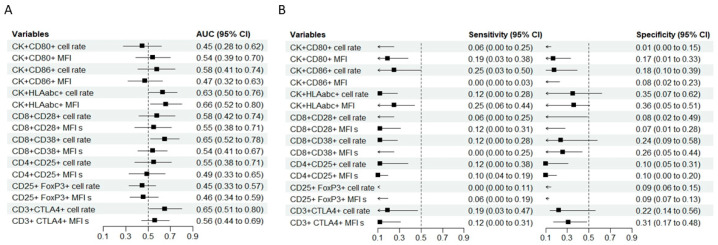
(**A**) The summary forest plot displays the area under the receiver operating characteristic curve (AUC) with a 95% confidence interval (CI) for each variable when predicting anastomotic leaks (prospective cohort). (**B**) The summary forest plots display the sensitivity (at 0.90 specificity) and specificity (at 0.90 sensitivity) with a 95% confidence interval (CI) for each variable when predicting the occurrence of anastomotic leaks (prospective cohort).

The discriminatory ability of single variables was limited (Figure 3). The CK^+^HLAabc^+^ MFI (AUC 0.66, 95% CI 0.52–0.80), CD8^+^CD38^+^ cell rate (AUC 0.65, 95% CI 0.52–0.78), and CD3^+^CTLA4^+^ cell rate (AUC 0.65, 95% CI 0.51–0.80) showed moderate predictive potential for ALs. When the specificity was fixed at 0.90, the sensitivity ranged from 0.00 (CK^+^CD86^+^ MFI, CD8^+^CD38^+^ MFI and CD25^+^ FoxP3^+^ cell rate) to 0.25 (CK^+^CD86^+^ cell rate and CK^+^HLAabc^+^ MFI); when the sensitivity was fixed at 0.90, the specificity ranged from 0.01 (CK^+^CD80^+^ cell rate) to 0.36 (CK^+^HLAabc^+^ MFI) (Figure 3).

### 3.2. Immune Microenvironment as Predictor of ALs

In the second step, we evaluated the role of the constitutive immune microenvironment in the histologically normal, tumor-adjacent rectal mucosa. A retrospective cohort of 262 patients was analyzed (165 males and 97 females; median age 66 years, IQR 55–76). ALs occurred in 35 patients (13.3%). The demographics and clinical characteristics are summarized in Table 1.

The absolute counts of T cell subpopulations measured by IHC did not differ significantly between patients with or without ALs (Table 3).

**Figure 4:**
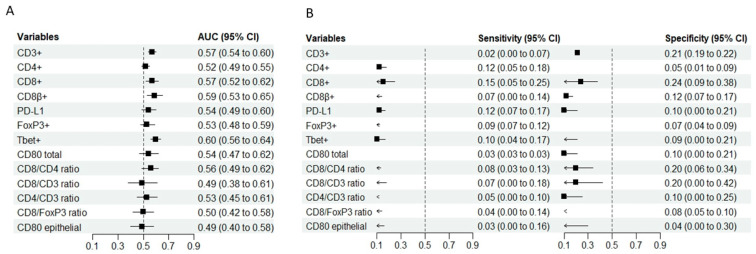
(**A**) The summary forest plot displays the area under the receiver operating characteristic curve (AUC) with a 95% confidence interval (CI) for each variable when predicting anastomotic leaks (retrospective cohort). (**B**) The summary forest plots display the sensitivity (at 0.90 specificity) and specificity (at 0.90 sensitivity) with a 95% confidence interval (CI) for each variable when predicting the occurrence of anastomotic leaks (retrospective cohort).

The AUC values were generally low (Figure 4). CD3^+^ (AUC 0.57, 95% CI 0.54–0.60), CD8^+^ (AUC 0.57, 95% CI 0.52–0.62), CD8β^+^ (AUC 0.59, 95% CI 0.53–0.65) and Tbet^+^ (AUC 0.60, 95% CI 0.56–0.64) showed some predictive ability for ALs. When the specificity was fixed at 0.90, the sensitivity ranged from 0.02 (CD3^+^) to 0.15 (CD8^+^); conversely, with the sensitivity fixed at 0.90, the specificity ranged from 0.04 (CD80 epithelial) to 0.24 (CD8^+^) (Figure 4). A sensitivity analysis of the immune microenvironment as predictor of ALs before multiple imputation is included in the Supplementary Materials (Supplementary Figures S2 and S3).

### 3.3. Clinical and Molecular Models to Predict ALs After Rectal Surgery

In the third step, we designed and compared predictive models combining constitutive immune predictors with clinical variables against a purely clinical model.

Model #1 (CD3^+^, CD8^+^, CD8β^+^ and Tbet^+^) achieved an AUC of 0.63 (95% CI 0.54–0.72), a sensitivity of 0.15 (95% CI 0.05–0.26) at a fixed specificity of 0.90 and a specificity of 0.16 (95% CI 0.04–0.29) at a fixed sensitivity of 0.90. Model #2 (CD3^+^, BMI, neutrophil/lymphocyte ratio and tumor location) yielded an AUC of 0.67 (95% CI 0.61–0.72), a sensitivity of 0.30 (95% CI 0.19–0.40) at a specificity of 0.90 and a specificity of 0.18 (95% CI 0.02–0.34) at a fixed sensitivity of 0.90. Model #3 (CD8^+^, BMI, neutrophil/lymphocyte ratio and tumor location) performed best at an AUC of 0.69 (95% CI 0.64–0.75), a sensitivity of 0.32 (95% CI 0.18–0.46) at a specificity of 0.90 and a specificity of 0.24 (95% CI 0.07–0.42) at a sensitivity of 0.90. Model #4 (CD8β^+^, BMI, neutrophil/lymphocyte ratio and tumor location) showed an AUC of 0.67 (95% CI 0.62–0.72), a sensitivity of 0.33 (95% CI 0.14–0.52) at a specificity of 0.90 and a specificity of 0.20 (95% CI 0.01–0.41) at a sensitivity of 0.90. Model #5 (Tbet^+^, BMI, neutrophil/lymphocyte ratio and tumor location) had an AUC of 0.68 (95% CI 0.60–0.76), a sensitivity of 0.33 (95% CI 0.17–0.50) at a fixed specificity of 0.90 and a specificity of 0.21 (95% CI 0.04- 0.37) at a fixed sensitivity of 0.90.

**Figure 5:**
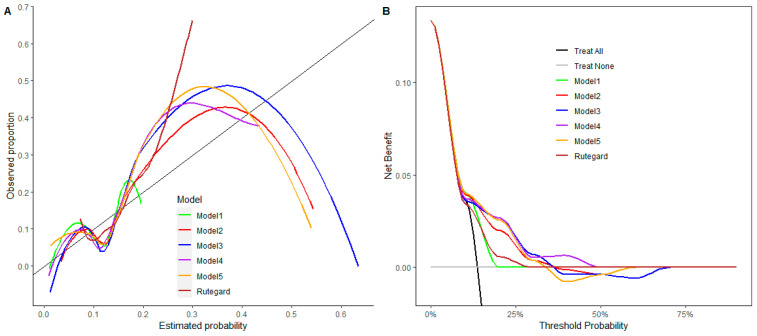
(**A**) The calibration curves show the correspondence between the model-estimated probability and the observed proportion. Departures from the diagonal line (showing perfect match between estimated and observed proportion) indicate worse calibration. (**B**) The decision curves display the net benefit of the risk prediction models compared with the two default strategies of treating all patients or treating none. A higher net benefit suggests higher clinical utility. Model 1 included CD3^+^, CD8^+^, CD8β^+^ and Tbet. Model 2 included CD3^+^, BMI, the neutrophil-to-lymphocyte ratio and tumor location. Model 3 included CD8^+^, BMI, the neutrophil-to-lymphocyte ratio and tumor location. Model 4 included CD8β^+^, BMI, the neutrophil-to-lymphocyte ratio and tumor location. Model 5 included Tbet, BMI, the neutrophil-to-lymphocyte ratio and tumor location. The model by Rutegard et al. [17] included male sex, BMI > 30 kg/m^2^ and radiotherapy.

Bootstrap internal validation (500 resampling) for models #2–5 confirmed the following AUCs: #2 had an AUC of 0.68 (95% CI 0.57–0.80), #3 had an AUC of 0.70 (95% CI 0.61–0.80), #4 had an AUC of 0.69 (95% CI 0.58–0.82), and #5 had an AUC of 0.70 (95% CI 0.58–0.82). For models #2–5, the sensitivity at a fixed specificity (0.90) ranged from 0.30 to 0.33, and the specificity at a fixed sensitivity of 0.90 ranged from 0.18 to 0.24. By comparison, the model by Rutegard et al. [17] (male sex, BMI > 30 kg/m^2^ and radiotherapy) achieved inferior performance, with an AUC of 0.61 (95% CI 0.53–0.69), a sensitivity of 0.06 (95% CI 0.06–0.06) at a specificity of 0.90 and a specificity of 0.00 (95% CI 0.00–0.00) at a sensitivity of 0.90. Calibration was suboptimal across all models (Figure 5A). Decision curve analysis showed modest clinical utility for AL selection at 15–30% thresholds, with model #4 offering the greatest net benefit and #1 offering the least (Figure 5B). A sensitivity analysis of model performance before multiple imputation is included in the Supplementary Materials (Supplementary Figure S4).

**Figure 6:**
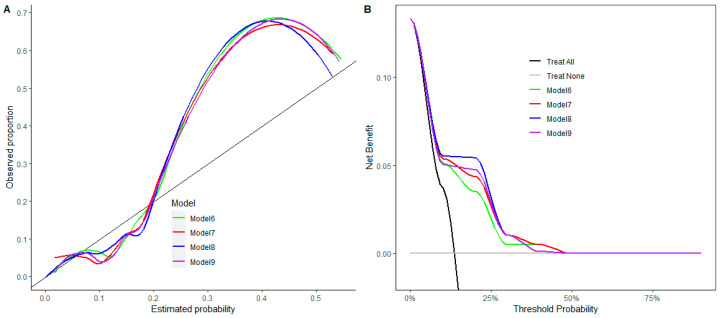
(**A**) The calibration curves show the correspondence between the model-estimated probability and the observed proportion. Departures from the diagonal line (showing perfect match between estimated and observed proportion) indicate worse calibration. (**B**) The decision curves display the net benefit of the risk prediction models compared with the two default strategies of treating all patients or treating none. A higher net benefit suggests higher clinical utility. Model 6 included CD3^+^, diabetes, distance from the anal verge and protective stoma. Model 7 included CD8^+^, diabetes, distance from the anal verge and protective stoma. Model 8 included CD8β^+^, diabetes, distance from the anal verge and protective stoma. Model 9 included Tbet, diabetes, distance from the anal verge and protective stoma.

Further models including diabetes, distance from the anal verge and protective stoma were developed as post hoc analyses with exploratory purposes. In the retrospective cohort, diabetes was present in 25 patients (9.5%), the median distance from the anal verge was 5 cm (IQR 4–7), and a protective stoma was created in 185 patients (70.6%). Model #6 (CD3^+^, diabetes, distance from the anal verge and protective stoma) had an AUC of 0.69 (95% CI 0.64–0.74), a sensitivity of 0.35 (95% CI 0.20–0.50) at a fixed specificity of 0.90 and a specificity of 0.20 (95% CI 0.11–0.28) at a fixed sensitivity of 0.90. Model #7 (CD8^+^, diabetes, distance from the anal verge and protective stoma) had an AUC of 0.70 (95% CI 0.58- 0.82), a sensitivity of 0.43 (95% CI 0.20–0.67) at a fixed specificity of 0.90 and a specificity of 0.24 (95% CI 0.10–0.38) at a fixed sensitivity of 0.90. Model #8 (CD8β^+^, diabetes, distance from the anal verge and protective stoma) had an AUC of 0.70 (95% CI 0.59–0.82), a sensitivity of 0.42 (95% CI 0.17–0.68) at a fixed specificity of 0.90 and a specificity of 0.23 (95% CI 0.05–0.42) at a fixed sensitivity of 0.90. Model #9 (Tbet^+^, diabetes, distance from the anal verge and protective stoma) had an AUC of 0.70 (95% CI 0.58–0.82), a sensitivity of 0.42 (95% CI 0.16–0.68) at a fixed specificity of 0.90 and a specificity of 0.22 (95% CI 0.05–0.39) at a fixed sensitivity of 0.90. Calibration remained suboptimal (Figure 6A). The decision curves showed modest clinical utility in selecting AL cases within the 15–30% threshold range, with Model #8 providing the greatest net benefit and Model #6 providing the least (Figure 6B).

## 4. Discussion

ALs remain the most fearsome and potentially devastating complication of colorectal surgery [24]. Unlike other types of complications, ALs can not only result in severe morbidity and mortality but also in an increase in the risk of local recurrence and a worsening prognosis [25,26,27,28]. Moreover, ALs can markedly impair a patient’s quality of life [4].

Numerous studies have studied risk factors for ALs. Penna et al. identified patient- related risk factors such as the male sex, obesity, smoking, diabetes, larger tumors (>25 mm) and a tumor height >4 cm from the anorectal junction upon MRI, while the only significant technical risk factor was excessive intraoperative blood loss >500 mL [13]. Qu et al.’s meta-analysis reported four intraoperative factors significantly associated with increased risk of ALs: prolonged operative time, more than two stapler firings, intraoperative transfusions or blood loss >100 mL and an anastomotic level <5 cm from the anal verge [29]. The STOMA score (18 factors) was developed to estimate the risk of 1-year stoma-free survival after an AL and showed good discrimination and calibration (c-index 0.71, 95% CI 0.66–0.76) [3].

While insufficient vascular supply has long been considered the main cause of ALs after rectal resection [6], the etiology is undoubtedly multifactorial. Conditions that alter the vasculature, such as smoking and diabetes, may increase the risk of ALs. In addition to vascular factors, local inflammation plays a crucial role in ALs. In recent years, increasing attention has been directed toward the role of danger-associated molecular patterns (DAMPs) and sterile inflammation in postoperative complications, including ALs [30]. DAMPs such as heath shock proteins, high-mobility group protein box 1 (HMGB1), extracellular adenosine triphosphate (ATP), mitochondrial DNA and fibronectin are released following surgical trauma, tissue hypoxia or extensive cell death [29,30,31,32]. Through binding to pattern recognition receptors, including Toll-like receptors (e.g., TLR4), DAMPs activate NF-κB signaling and inflammasome pathways, particularly NLRP3, leading to the production of pro-inflammatory cytokines such as IL-1, IL-6, IL-12 and TNF-α. [30,33]. Although traditionally studied in innate immune cells, these pathways are also crucial for T cell activation and effector function. Given that the intestinal mucosa contains the largest lymphoid compartment in the human body, dysregulation of mucosal immune homeostasis may critically impair tissue repair and barrier integrity [34].

Within this biological framework, the IMMUNOREACT 4 study explored whether immune activation in histologically normal, tumor-adjacent rectal mucosa predisposes patients to ALs. In the prospective cohort analyzed via flow cytometry, patients who developed ALs exhibited significantly higher expression of epithelial HLA class I. CK^+^HLAabc^+^ MFI increased the CD8^+^CD38^+^ and CD3^+^CTLA4^+^ cell rates. These findings suggest a state of heightened epithelial–immune interaction, characterized by cytotoxic T cell activation modulated by immune checkpoint signaling in patients predisposed to ALs. Immunohistochemistry in the retrospective cohort further supposed this biological signal, showing higher counts of CD3^+^, CD8^+^, CD8β^+^ and Tbet^+^ cells in patients who developed ALs. These markers reflect cytotoxic and Th1-polarized immune responses, which may represent downstream effectors of DAMP-driven inflammasome activation. Although the absolute differences were modest, and predictive performance was limited, the convergence of findings across two independent analytical platforms strengthens the biological plausibility of our observations. The precise trigger of this immune activation remains speculative. DAMPs such as HMGB1 and HSP70, released from dying or hypoxic cells, may promote dendritic cells maturation with upregulation of HLA on its surface [35]. A similar process may occur in epithelial cells themselves, which can function as non-professional antigen-presenting cells. DAMP-induced epithelial activation may enhance HLA class I expression, promoting cytotoxic T cell engagement. Moreover, DAMPs can induce sterile auto-inflammation via innate immunity pathways and upregulation of HLA molecules presenting self-peptides or stress-associated ligands [34]. These mechanisms, while not directly tested in the present study, provide a biologically coherent hypothesis linking tumor-conditioned mucosal immune activation to impaired anastomotic healing. Importantly, these interpretations should be regarded as hypothesis-generating, given the observational nature of the study.

Notably, 57.1% of the patients in the prospective cohort and 43.3% in the retrospective cohort received neoadjuvant treatment, which is known to profoundly modulate the peritumoral immune microenvironment [35]. Several studies have demonstrated that neoadjuvant radiotherapy (RT) and chemoradiotherapy (CRT) increase overall T cell infiltration (CD3^+^) in rectal cancer tissue, though short-course RT yields lower CD3^+^ densities than long-course regimens, depending on fractionation and the interval to surgery. Cytotoxic CD8^+^ T cells are particularly sensitive: CRT boosts their density and activity (e.g., granzyme B upregulation), especially in good responders, with delayed surgery enhancing accumulation compared with immediate post-short-course RT [36]. Th1-associated T helper cells (Tbet^+^) show early depletion post-RT, followed by repopulation if surgery is delayed. CRT shifts the balance toward CD8^+^ predominance, reducing T helper and regulatory T cells [36]. Taken together, these observations indicate that neoadjuvant therapy acts as a dynamic immune modulator, initially inducing immune depletion and tissue injury and subsequently promoting a more homogeneous and functionally active cytotoxic immune infiltrate. Therefore, the immune profiles observed in the present study likely reflect the combined effects of tumor-conditioned field biology and treatment-induced immune remodeling. Importantly, this interaction does not weaken our central hypothesis but rather supports the concept that neoadjuvant therapy may unmask or amplify preexisting mucosal immune vulnerability at the intended anastomotic site, thereby contributing to impaired healing in susceptible patients.

In the second IMMUNOREACT 4 step, we evaluated whether the immune features of tumor-adjacent mucosa could contribute to exploratory predictive models of ALs. Immunohistochemistry was selected as the method of choice given its widespread availability, standardization and feasibility in routine pathology practice. Among the markers assessed, CD3^+^, CD8^+^, CD8β^+^ and Tbet^+^ showed limited but consistent predictive value. These immune markers likely reflect preexisting mucosal vulnerability due to hypoxia, subclinical inflammation, immune dysregulation or microbial translocation at the intended anastomotic site [37]. Consistent with our previous study, our findings reinforce the central role of activated CD8^+^ T cells within the rectal mucosa as potential predictors of ALs [38]. Notably, our findings raise the possibility that preoperative assessment of immune markers on diagnostic biopsies could help identify patients at higher risk of anastomotic leak. Such information could, in principle, inform surgical strategies. Nevertheless, the differences between preoperative biopsies and tissue sampled intraoperatively represent important limitations that require careful validation in future prospective studies.

In the third step, we aimed to design and compare the accuracy of different models including different constitutive immune predictors and compare them to a pure clinical model to predict ALs. The model that included only local immune markers obtained poor accuracy in predicting ALs as well as that with only clinical predictors. Therefore, we explored different combinations of clinical predictors and immunological markers. Due to insufficient data, our study was limited in that it did not consider certain important intraoperative variables such as the surgeon’s skill and experience and intraoperative blood assessment with indocyanine green (ICG). Model #3 (CD8^+^, BMI, neutrophil-to-lymphocyte ratio and tumor location) and model #4 (CD8β^+^, BMI, neutrophil/lymphocyte ratio and tumor location) had the best accuracy, being about 70%. Similar models including diabetes, the distance from the anal verge and protective stoma as clinical covariates substantially confirmed those findings on immunological markers. These results should be interpreted in light of the retrospective design of the study and the relatively low sample size, which inevitably limited the number and complexity of the models that could be developed. The inclusion of only Italian centers further restricts the generalizability of the findings to similar settings. Moreover, the missing data in molecular predictors were handled using multiple imputation, but the amount of missing information suggests caution in interpretation of the findings. Hence, the predictive models were developed for exploratory purposes and aimed at identifying tissue-level biological vulnerability rather than at providing clinically actionable risk prediction tools. Furthermore, the suboptimal calibration of the models could be due to the small number of AL cases and the absence of unknown important predictors in the models. Future prospective studies may build on these considerations to improve the clinical utility of the modes. However, these apparently modest results hold relevance for several reasons. First, similar models based solely on clinical predictors, such as that by Rutegard et al. [17], reported considerably lower accuracy than our two models. Second, it is important to recognize the context in which these models operate. An AL is an outcome that surgeons actively strive to prevent through multiple perioperative strategies (i.e., selective avoidance of anastomosis in patients at risk, diverting ileostomy, intraoperative assessment of vascular perfusion with indocyanine green and preoperative steroid tapering). These preventive interventions inevitably reduce the number of observable events, thereby contributing to the apparent “missing” 30% in predictive accuracy. Thus, our models focused on ALs that occur despite the implementation of current preventive measures rather than every possible AL, in line with all prior work in this field. A mechanistic validation of our findings (i.e., using mouse models) would have been useful, but this was beyond the aims of the current study, and it will be the object of a further study.

## 5. Conclusions

In conclusion, this multicenter translational study demonstrates that immune activation within histologically normal, tumor-adjacent rectal mucosa is associated with the development of ALs after rectal cancer surgery. Increased epithelial HLA class I expression and enrichment of cytotoxic and Th1-polarized T cell populations suggest the presence of a tumor-conditioned immune field that may compromise anastomotic healing.

Although the predictive performance of the proposed clinical–immune models remains modest, and calibration is suboptimal, combining immune microenvironmental features with clinical variables improved discrimination compared with purely clinical models.

These results should be interpreted as hypothesis-generating rather than immediately clinically actionable. Further prospective studies, external validation and integration with additional biological layers are required to determine whether mucosal immune profiling can contribute to individualized strategies for the prevention of anastomotic leaks.

## Figures and Tables

**Table 1:** Demographics and clinical characteristics of patients who underwent radical surgery for rectal cancer with colorectal anastomosis: prospective cohort and retrospective cohort.

Variables	Prospective Cohort (n = 121)	Retrospective Cohort (n = 262)
Age (years)	65 (52–75) ^a^	66 (55–76)
Males	71 (58.7%)	165 (63.0%)
BMI, kg/m^2^	25.3 (23–0-28.6) ^b^	25.1 (22.6–27.4) ^c^
Grading		
0	13/92 (14.1%)	5/212 (2.3%)
1	27/92 (29.4%)	22/212 (10.4%)
2	30/92 (32.6%)	142/212 (67.0%)
3	21/92 (22.8%)	43/212 (20.3%)
4	1/92 (1.1%)	0/212 (0.0%)
T stage		
0	19/115 (16.5%)	28/261 (10.7%)
1	19/115 (16.5%)	31/261 (11.9%)
2	30/115 (26.1%)	78/261 (29.9%)
3	43/115 (37.4%)	103/261 (39.5%)
4	4/115 (3.5%)	21/261 (8.0%)
N^+^ stage	33/115 (28.7%)	99/260 (38.1%)
M^+^ stage	4/116 (3.4%)	31/255 (12.1%)
Neoadjuvant therapy	68/119 (57.1%)	113/261 (43.3%)
Response to neoadjuvant therapy		
Complete	11 (16.2%)	22 (19.5%)
Major	57 (83.8%)	91 (80.5%)
Cancer site		
Rectum < 15 cm form anal verge	103/116 (88.8%)	187/229 (81.7%)
Rectosigmoid junction	13/116 (11.2%)	42/229 (18.3%)
Anastomosis type		
Colo-anal	15/121 (12.4%)	26/261 (10.0%)
Colo-rectal	101/121 (83.5%)	230/261 (88.1%)
Ileo-anal	5/121 (4.1%)	5/261 (1.9%)
Protective stoma creation	81/121 (66.9%)	185/262 (70.6%)
Transanal drainage placed to protect the anastomosis	Nil	Nil
Anastomotic leak	16 (13.2%)	35 (13.3%)

Data are summarized as n (%) or median (IQR). Data were not available in ^a^ 4, ^b^ 17 and ^c^ 72 patients.

**Table 2:** Activation profile of epithelial cells and intramucosal T lymphocytes stratified by occurrence of anastomotic leaks (prospective cohort).

Variables	Patients with ALs (n = 16)	Patients Without ALs (n = 105)	*p* Value
CK^+^CD80^+^ cell rate	14 (11–51)	17 (8–31)	0.51
CK^+^CD80^+^ MFI	126 (107–166)	123 (99–176)	0.58
CK^+^CD86^+^ cell rate	18 (8–33)	15 (7–27)	0.33
CK^+^CD86^+^ MFI	124 (88–162)	117 (94–167)	0.72
CK^+^HLAabc^+^ cell rate	62 (45–68)	37 (15–78)	0.10
CK^+^HLAabc^+^ MFI	546 (445–719)	407 (247–642)	0.04
CD8^+^CD28^+^ cell rate	4 (2–6)	2 (1–5)	0.30
CD8^+^CD28^+^ MFI	34 (27–59)	42 (30–58)	0.53
CD8^+^CD38^+^ cell rate	8 (4–10)	4 (2–8)	0.05
CD8^+^CD38^+^ MFI	46 (39–55)	48 (37–66)	0.63
CD4^+^CD25^+^ cell rate	2 (0–5)	1 (0–3)	0.56
CD4^+^CD25^+^ MFI	18 (0–41)	18 (0–32)	0.91
CD25^+^ FoxP3^+^ cell rate	0 (0–0)	0 (0–2)	0.45
CD25^+^ FoxP3^+^ MFI	0 (0–213)	0 (0–324)	0.57
CD3^+^CTLA4^+^ cell rate	8 (3–15)	3 (1–10)	0.04
CD3^+^ CTLA4^+^ MFI	33 (26–48)	31 (17–54)	0.41

Data are summarized as median (IQR). AL = anastomotic leak. MFI = mean fluorescence intensity.

**Table 3:** Expression profile in the retrospective cohort stratified by occurrence of anastomotic leaks (retrospective cohort).

Variables	Missing Information (%)	Patients with ALs (n = 35)	Patients Without ALs (n = 227)	*p* Value
CD3^+ a^	8.4%	106 (79–126)	121 (74–159)	0.45
CD4^+ b^	17.5%	36 (28–44)	37 (28–44)	0.77
CD8^+ c^	12.2%	31 (25–37)	35 (25–43)	0.10
CD8β^+ d^	19.5%	1.5 (1.0–2.4)	2.0 (1.0–4.0)	0.05
PD-L1 ^e^	18.7%	1.0 (1.0–1.5)	1.0 (1.0–1.5)	0.62
FoxP3^+ f^	24.0%	4.0 (3.0–5.5)	4.7 (3.0–6.0)	0.37
Tbet^+ g^	21.7%	2.5 (1.0–3.0)	3.1 (2.0–5.0)	0.05
CD80 total ^h^	23.7%	74 (52–88)	65 (48–81)	0.37
CD8/CD4 ratio ^i^	25.6%	0.8 (0.7–0.9)	0.9 (0.7–1.1)	0.36
CD8/CD3 ratio ^j^	17.2%	0.3 (0.2–0.4)	0.3 (0.2–0.4)	0.44
CD4/CD3 ratio ^k^	22.1%	0.3 (0.3–0.5)	0.3 (0.2–0.5)	0.64
CD8/FoxP3 ratio ^l^	31.7%	7.6 (3.9–9.7)	6.4 (4.5–9.6)	0.98
CD80 epithelial ^m^	32.1%	1.0 (0.2–1.0)	0.5 (0.0–1.0)	0.61
PMS2^+^: ^g^ 1 2	21.7%	0/30 (0.0%) 30/30 (100.0%)	2/175 (1.1%) 173/175 (98.9%)	0.99
MSH6^+^: ^n^ 1 2	21.0%	0/31 31/31	5/176 171/176	0.99

Data are summarized as n (%) or median (IQR). Data were not available in ^a^ 22, ^b^ 46, ^c^ 32, ^d^ 51, ^e^ 49, ^f^ 63, ^g^ 57, ^h^ 62, ^i^ 67, ^j^ 45, ^k^ 58, ^l^ 83, ^m^ 84 and ^n^ 55 patients.

## Data Availability

The anonymized clinical and immunophenotyping datasets analyzed in this study are available from the corresponding author upon reasonable request. Patient confidentiality regulations prevent public submission of raw clinical data.

## Supplementary Materials

The following supporting information can be downloaded at https://www.mdpi.com/article/10.3390/cancers18040571/s1. Table S1. Immunohistochemistry antibodies used in the study. Table S2. Flow cytometry antibodies used in the study. Figure S1. Density plots of the expression profile (CD3^+^, CD4^+^, CD8^+^, CD8β^+^, PD-L1, FoxP3^+^, Tbet^+^, CD80 tota, CD8/CD4 ratio, CD8/CD3, CD4/CD3 ratio, CD8/FoxP3 ratio, CD80 epithelial) in the retrospective cohort after multiple imputation. The plots show that imputed and observed data match up well, indicating the plausibility of imputations. Figure S2. Sensitivity analysis before multiple imputation: the summary forest plot displays the area under the receiver operating characteristic curve (AUC) with 95% confidence interval (CI) for each variable when predicting anastomotic leak (retrospective cohort). Figure S3. Sensitivity analysis before multiple imputation: the summary forest plots display sensitivity (at 0.90 specificity) and specificity (at 0.90 sensitivity) with 95% confidence interval (CI) for each variable when predicting the occurrence of anastomotic leak (retrospective cohort). Figure S4. Sensitivity analysis before multiple imputation: A: The calibration curves show the correspondence between model-estimated probability and observed proportion; departures from the diagonal line indicate worse calibration. B: The decision curves display the net benefit of the risk prediction models compared to the two default strategies of treating all patients or treating none; higher net benefit suggests higher clinical utility. Model 1 included CD3^+^, CD8^+^, CD8β^+^, Tbet. Model 2 included CD3^+^, BMI, neutrophil to lymphocyte ratio, tumor location. Model 3 included CD8^+^, BMI, neutrophil to lymphocyte ratio, tumor location. Model 4 included CD8β^+^, BMI, neutrophil to lymphocyte ratio, tumor location. Model 5 included Tbet, BMI, neutrophil to lymphocyte ratio, tumor location. The model by Rutegard et al. [17] included male sex, BMI > 30 kg/m^2^ and radiotherapy.
