# Supplementary material for: IMMUNOREACT 4: Peritumoral Microenvironment Associated with Anastomotic Leaks After Surgery for Rectal Cancer
Source: Cancers (Basel). 2026 Feb 9;18(4):571. doi: 10.3390/cancers18040571 (PMC12938832; doi:10.3390/cancers18040571)
Supplement: Supplementary file 1 [file cancers-18-00571-s001.zip › cancers-4088579-supplementary.pdf]

**Supplementary Material**

*IMMUNOREACT project*

The first part of the IMMUNOREACT project was focused on patients with early rectal cancer who did not have any neoadjuvant therapy aiming to detect immunological predictors of local extension in the healthy rectal mucosa (IMMUNOREACT 1, clinicaltrials.gov NCT04915326). The second part of the study was focused on patients with rectal cancer after neoadjuvant therapy aiming to detect immunological predictors of local extension in the healthy rectal mucosa (IMMUNOREACT 2, clinicaltrials.gov NCT04915326).

*Clinical data*

Clinical data included patient-related variables (sex, age, BMI greater than 30 kg/m2, weight loss, and smoking habits), disease-related variables (cancer staging) and treatment-related variables (center case volume, type of resection, open vs. laparoscopic approach, intraoperative contamination, anastomotic level, type of anastomosis, stoma construction, blood transfusions, and neoadjuvant therapy). The minimum follow-up period required to define AL was 30 days after rectal resection or after stoma closure in those patients with a temporary stoma. To assess the gravity of AL the classification described by Soeters et al. [1] was used which includes four progressive severity grades.

*Surgical techniques*

In this multicentric study we included patients who had a straight colorectal anastomosis for gastrointestinal reconstruction after low anterior resection of rectal cancer (double purse-string circular stapled anastomosis or the single stapling technique, or the conventional double stapling technique).

**Supplementary Table S1. Immunohistochemistry antibodies used in the study**

| Target | Clone | Dilution | Manufacturer |
|---|---|---|---|
| CD3 | LN10 | 1:100 | Leica Biosystems |
| CD4 | 4B12 | 1:100 | Leica Biosystems |
| CD8 | 4B11 | 1:100 | Leica Biosystems |
| CD8beta | 039 | 1:200 | Sino Biological |
| Tbet | 39D | 1:200 | Abcam |
| FOXP3 | 236A/E7 | 1:100 | Abcam |
| PD-L1 | EPR1161(2) | 1:50 | Abcam |

**Supplementary Table S2. Flow cytometry antibodies used in the study**

| Target | Conjugate | Clone | Company |
|---|---|---|---|
| CD3 | APC | OKT3 | eBioscience |
| CD4 | PE-Cy7 | RPA-T4 | eBioscience |
| CD8a | PE | HIT8a | eBioscience |
| CD25 | PE | BC96 | eBioscience |
| CD28 | FITC | CD28.2 | eBioscience |
| CD38 | FITC | HIT2 | eBioscience |
| CD80 | FITC | 2D10.4 | eBioscience |
| CD86 | AlexaFluor 488 | IT2.2 | eBioscience |
| CTLA-4 | PE | L3D10 | Biolegend |
| FoxP3 | AlexaFluor 488 | 150D/E4 | eBioscience |
| HLAabc | FITC | W6/32 | eBioscience |
| Pan-Cytokeratin | PE | C-11 | Abcam |

**Supplementary Figure S1**. Density plots of the expression profile (CD3+, CD4+, CD8+, CD8β+, PD-L1, FoxP3+, Tbet+, CD80 tota, CD8/CD4 ratio, CD8/CD3, CD4/CD3 ratio, CD8/FoxP3 ratio, CD80 epithelial) in the retrospective cohort after multiple imputation. The plots show that imputed and observed data match up well, indicating the plausibility of imputations.

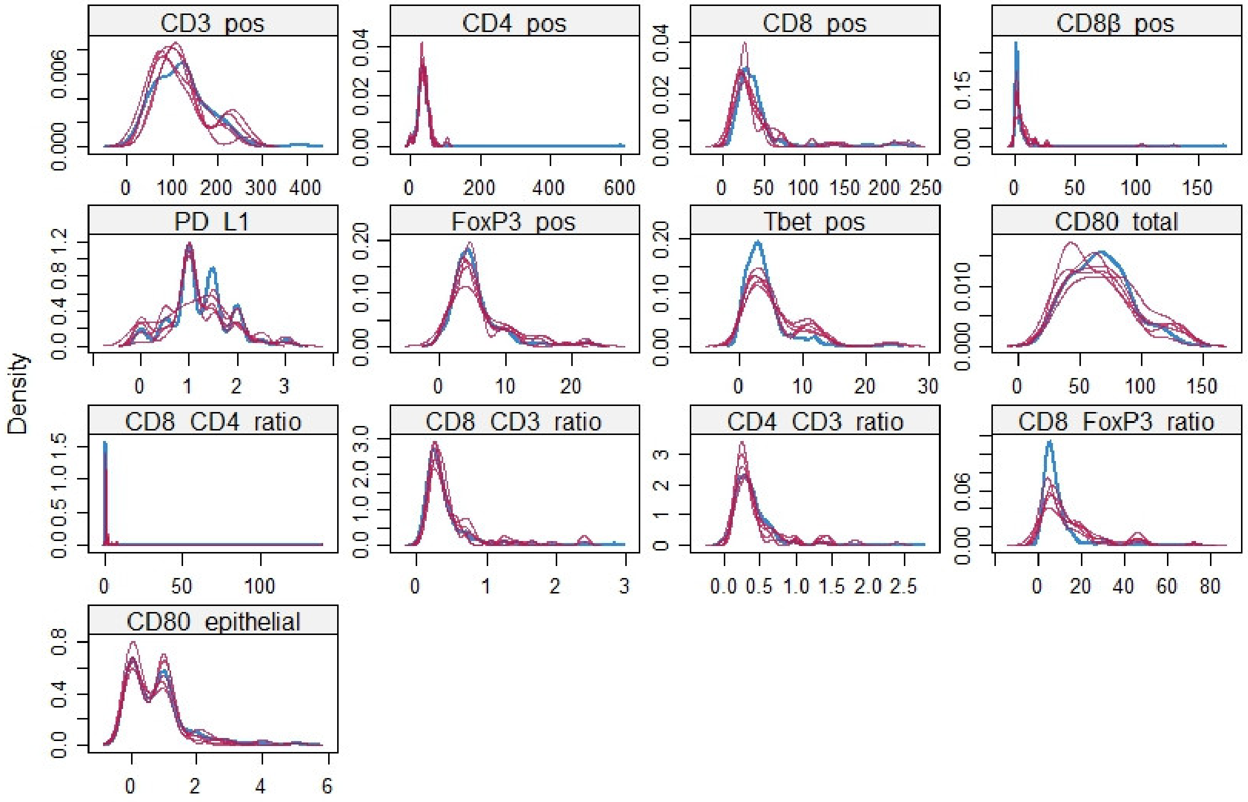

**Supplementary Figure S2**. Sensitivity analysis before multiple imputation: the summary forest plot displays the area under the receiver operating characteristic curve (AUC) with 95% confidence interval (CI) for each variable when predicting anastomotic leak (retrospective cohort).

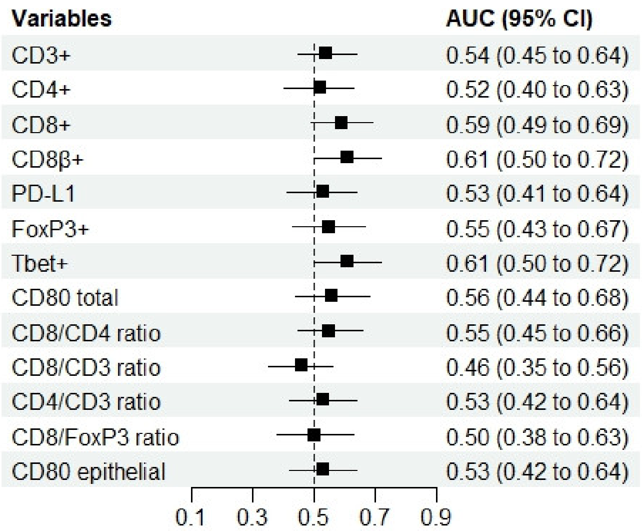

**Supplementary Figure S3**. Sensitivity analysis before multiple imputation: the summary forest plots display sensitivity (at 0.90 specificity) and specificity (at 0.90 sensitivity) with 95% confidence interval (CI) for each variable when predicting the occurrence of anastomotic leak (retrospective cohort).

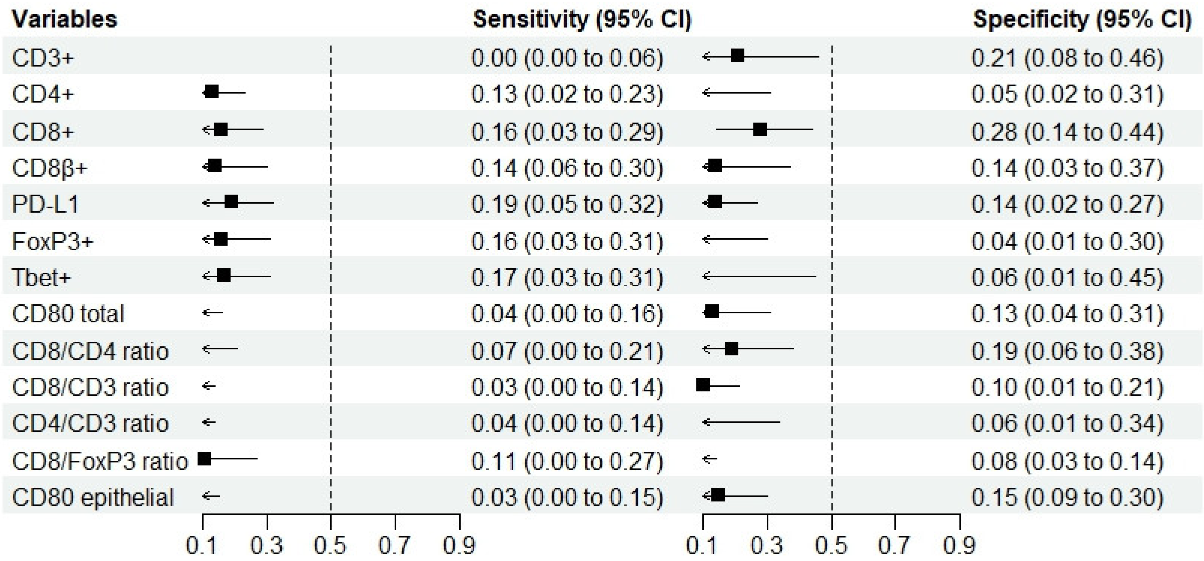

| Variables | Sensitivity (95% CI) | Specificity (95% CI) |
|---|---|---|
| CD3+ | 0.00 (0.00 to 0.06) | 0.21 (0.08 to 0.46) |
| CD4+ | 0.13 (0.02 to 0.23) | 0.05 (0.02 to 0.31) |
| CD8+ | 0.16 (0.03 to 0.29) | 0.28 (0.14 to 0.44) |
| CD8β+ | 0.14 (0.06 to 0.30) | 0.14 (0.03 to 0.37) |
| PD-L1 | 0.19 (0.05 to 0.32) | 0.14 (0.02 to 0.27) |
| FoxP3+ | 0.16 (0.03 to 0.31) | 0.04 (0.01 to 0.30) |
| Tbet+ | 0.17 (0.03 to 0.31) | 0.06 (0.01 to 0.45) |
| CD80 total | 0.04 (0.00 to 0.16) | 0.13 (0.04 to 0.31) |
| CD8/CD4 ratio | 0.07 (0.00 to 0.21) | 0.19 (0.06 to 0.38) |
| CD8/CD3 ratio | 0.03 (0.00 to 0.14) | 0.10 (0.01 to 0.21) |
| CD4/CD3 ratio | 0.04 (0.00 to 0.14) | 0.06 (0.01 to 0.34) |
| CD8/FoxP3 ratio | 0.11 (0.00 to 0.27) | 0.08 (0.03 to 0.14) |
| CD80 epithelial | 0.03 (0.00 to 0.15) | 0.15 (0.09 to 0.30) |

**Supplementary Figure S4**. Sensitivity analysis before multiple imputation: A: The calibration curves show the correspondence between model-estimated probability and observed proportion; departures from the diagonal line indicate worse calibration. B: The decision curves display the net benefit of the risk prediction models compared to the two default strategies of treating all patients or treating none; higher net benefit suggests higher clinical utility. Model 1 included CD3+, CD8+, CD8β+, Tbet. Model 2 included CD3+, BMI, neutrophil to lymphocyte ratio, tumor location. Model 3 included CD8+, BMI, neutrophil to lymphocyte ratio, tumor location. Model 4 included CD8β+, BMI, neutrophil to lymphocyte ratio, tumor location. Model 5 included Tbet, BMI, neutrophil to lymphocyte ratio, tumor location. The model by Rutegard et al. [17] included male sex, BMI>30 kg/m2 and radiotherapy.

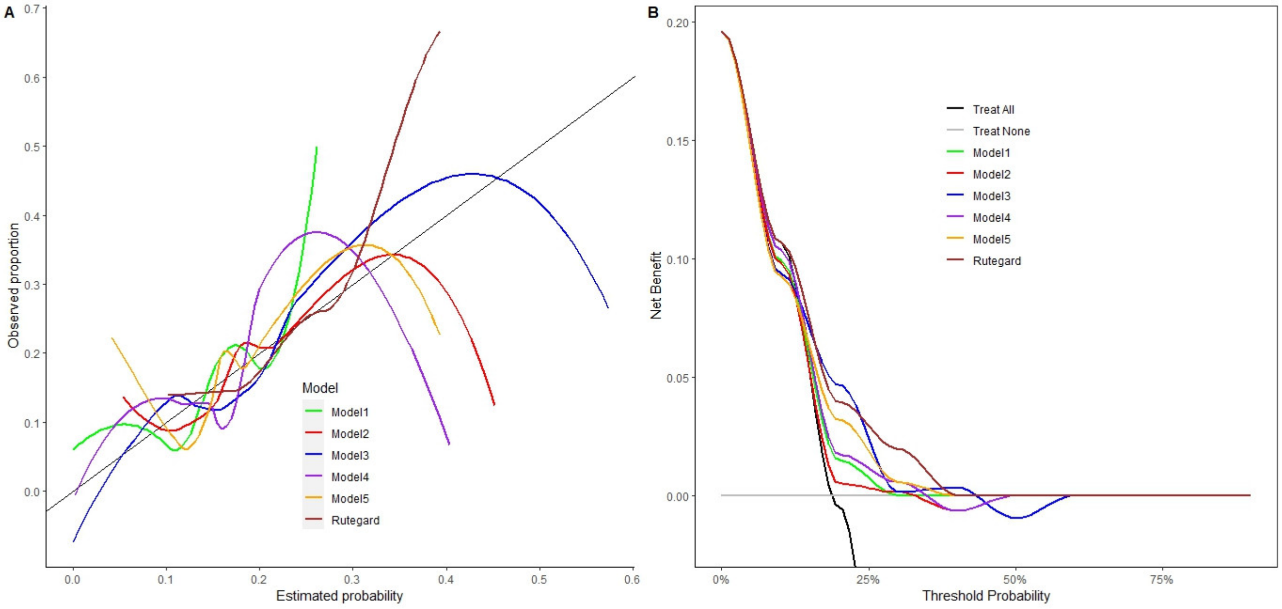


References

1. Soeters PB, de Zoete JP, Dejong CH, Williams NS, Baeten CG. Colorectal surgery and anastomotic leakage. Dig Surg. 2002;19(2):150-5. doi: 10.1159/000052031.
